# Medical imaging in immunotherapy response evaluation: from RECIST to AI-driven longitudinal models, and the distinction between risk prediction and kinetic diagnosis

**DOI:** 10.3389/fmed.2026.1954076

**Published:** 2026-09-07

**Authors:** Huzhe Cui, Zhengri Piao, Songnan Zhang

**Affiliations:** 1 Department of Radiology, The Affiliated Hospital of Yanbian University (Yanbian Hospital), Yanji, China; 2 Department of Radiation Oncology, The Affiliated Hospital of Yanbian University (Yanbian Hospital), Yanji, China; 3 Department of Oncology, The Affiliated Hospital of Yanbian University (Yanbian Hospital), Yanji, China

**Keywords:** artificial intelligence, hyperprogression, immunotherapy, medical imaging, pseudoprogression, radiomics

## Abstract

Immune checkpoint inhibitors have reshaped cancer treatment, but the response patterns they produce—pseudoprogression, dissociated responses, and hyperprogressive disease (HPD)—fit awkwardly into the size-based logic of RECIST 1.1. This narrative review, prepared with reference to the SANRA criteria, follows the evolution of response assessment from RECIST 1.1 through immune-adapted criteria such as iRECIST to functional and molecular imaging and, most recently, artificial intelligence (AI) models trained on longitudinal imaging. One distinction runs through the entire argument: current AI and radiomics models are risk-stratification tools, not diagnostic classifiers of HPD. Because HPD is defined as acceleration of tumor growth relative to a pretreatment trajectory, its diagnosis requires at least three imaging time points (pre-baseline, baseline, and first on-treatment assessment); no single-time-point model, however sophisticated, can reconstruct that trajectory. We appraise Image Biomarker Standardisation Initiative-compliant radiomics, convolutional neural networks, vision transformers, and early longitudinal modeling, and we compare early, joint, and late strategies for fusing imaging with clinical and molecular data. The clinical meaning of reported performance gains is weighed against standard assessment practice. Most evidence remains retrospective, and persistent obstacles include heterogeneous acquisition protocols, small cohorts, missing pre-baseline scans, endpoint misclassification, weak external validation, and limited biological interpretability. Prospective multicenter data collection, transparent model reporting, calibration and domain-shift testing, and biologically anchored validation are prerequisites before imaging-based AI biomarkers can enter routine immuno-oncology care.

## 1 Introduction

Immune checkpoint inhibitors now anchor the treatment of many advanced cancers, working by releasing the brakes that tumors place on host T cells (1). The same biology that produces durable responses also produces response patterns that a caliper-based reading of CT scans was never designed to capture. Beyond pseudoprogression—the early radiographic enlargement that later melts away—clinicians must contend with hyperprogressive disease (HPD), a striking acceleration of growth after treatment starts, and with dissociated responses, in which some lesions shrink while others grow. Misclassifying any of these has immediate consequences: stopping a drug that is actually working, or continuing one that is not.

Pseudoprogression reflects immune-cell infiltration and edema rather than tumor proliferation. Its pooled incidence across solid tumors is roughly 6% (95% CI 5%–7%), with estimates by tumor type and definition spanning about 5%–7% (2). HPD is rarer but carries a distinctly worse prognosis (3); whether it is a genuine drug-induced phenomenon or simply the recognizable tail of very aggressive tumors remains an open question, and several authors have argued that the term may describe natural history rather than a treatment effect (3). Dissociated responses affect roughly 3% of patients on combination immunotherapy and expose the spatial heterogeneity of the tumor immune microenvironment (4). When response is judged by the sum of target-lesion diameters alone, all of this nuance is averaged away.

The RECIST working group answered some of these gaps with iRECIST in 2017 (5), the latest step in a lineage that began with the immune-related response criteria (irRC) in 2009 and irRECIST in 2013 (6). By adding the category of unconfirmed progressive disease and mandating a confirmatory scan 4–8 weeks later, iRECIST built a safety net against stopping therapy during what later turns out to be pseudoprogression (5). The net catches real patients: in a pooled analysis of 1,765 patients treated with avelumab across two trials, 8.3% had RECIST 1.1-defined progressive disease as their best overall response yet achieved immune-related disease control when the same scans were re-read with irRECIST (6). Even so, iRECIST remains a morphologic instrument. It reads diameters; it does not read biology, and it is not designed to detect the immunobiological shifts that precede radiographic change.

**Figure 1:**
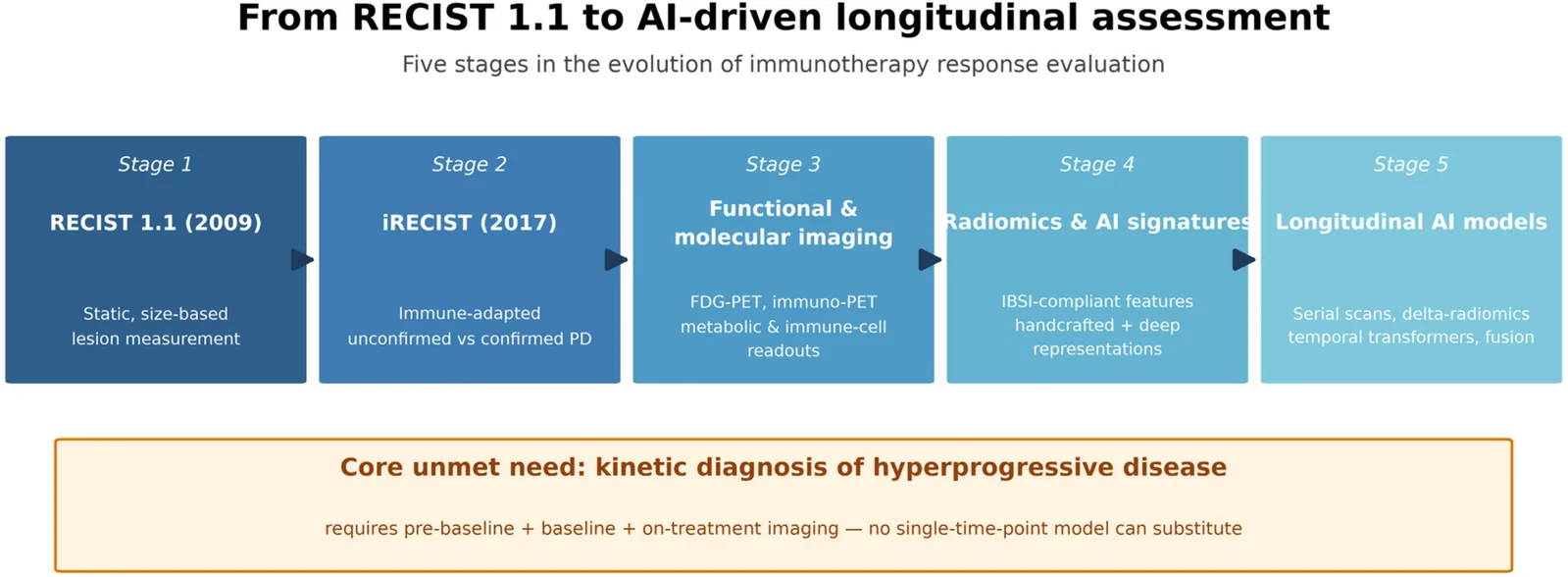
Evolution of immunotherapy response assessment from static, size-based criteria (RECIST 1.1) through immune-adapted criteria (iRECIST), functional and molecular imaging, radiomics-based signatures, and longitudinal AI models. The shaded bar highlights the core unmet need: kinetic diagnosis of hyperprogressive disease requires a pre-baseline imaging time point that single-time-point models cannot replace.

Against this background, this review does three things. It traces the evolution of response assessment from RECIST 1.1 through immune-adapted criteria to functional and molecular imaging and, most recently, AI models trained on longitudinal imaging (Figure 1). It asks what the published performance figures of these models actually support. And it keeps in view a distinction that is easy to blur: retrospective models that flag patients at risk of hyperprogression (7) are not, by that fact, diagnosing HPD. A kinetic diagnosis requires a pretreatment growth trajectory; a single-time-point risk score does not provide one.

## 2 Review methodology

This narrative review was planned and reported with reference to the SANRA criteria (8). PubMed/MEDLINE was searched through July 19, 2026, using Boolean combinations of four thematic term groups: (i) response criteria (RECIST, iRECIST, irRECIST, immune-related response criteria); (ii) atypical response patterns (pseudoprogression, hyperprogression, tumor growth rate, dissociated response); (iii) imaging modalities (CT, MRI, FDG PET, immuno-PET, radiomics, delta-radiomics); and (iv) AI methods (deep learning, convolutional neural network, vision transformer, longitudinal imaging, multimodal fusion, domain adaptation, explainable AI). Reference lists of eligible articles were additionally screened (snowballing) for further sources.

Inclusion criteria were English-language original research in humans, consensus guidelines, systematic reviews and meta-analyses, and multicenter studies with explicit methodological reporting. Preclinical studies were included only when they introduced emerging tracers or network architectures not yet tested in patients. Exclusion criteria were non-English publications; case reports and small case series, except where a response pattern was first defined; conference abstracts without full text; and non-peer-reviewed preprints, which were not used to support quantitative claims. Titles and abstracts were screened by one reviewer; disagreements were resolved by discussion with a second reviewer, and potentially eligible records were assessed in full text. No dual independent screening and no quantitative synthesis were performed; this work is therefore a narrative, not a systematic, review and was not registered. AI studies were appraised against the CLAIM 2024 checklist (9), the TRIPOD + AI statement (10), and the PROBAST + AI tool (11). Sixty sources were included in the final reference list.

## 3 Evolution of immunotherapy response criteria and imaging challenges

### 3.1 From RECIST to iRECIST: rationale, limitations, and operational barriers

RECIST 1.1 rests on a linear premise: shrinking tumors signal drug efficacy. In the chemotherapy era this worked well enough; checkpoint inhibitors exposed the blind spots (1). A real-world study of melanoma and non-small-cell lung cancer (NSCLC) found that 33.6% of patients labeled progressive disease (PD) by RECIST 1.1 did not meet iRECIST criteria for confirmed progression—in other words, roughly one patient in three might have stopped an effective therapy prematurely under the older rules (12).

iRECIST separates unconfirmed progression (iUPD) from confirmed progression (iCPD) and requires a 4–8-week confirmatory scan (5). The framework performs as intended in trials: in a KEYNOTE-002 cohort of advanced melanoma, 14 of 78 patients (17.9%) who continued treatment and imaging beyond RECIST 1.1-defined PD turned out to have pseudoprogression, and these patients lived markedly longer than those with true progression (median overall survival 29.9 vs. 8.0 months) (13). Similar patterns were reported in MSI-H/dMMR colorectal cancer treated with nivolumab plus ipilimumab (14) and across a range of atypical response trajectories (15). The catch is that the benefit is measurable but small: a systematic review and meta-analysis found only a marginal gain in restricted mean survival time (0.46 months) and no significant difference in objective response or disease-control rates between RECIST 1.1 and iRECIST assessment (16).

The confirmatory-scan pathway also carries costs that the framework itself does not price in. A substantial share of patients deteriorate clinically before the scheduled reassessment and never receive the confirmatory scan; attrition between iUPD and iCPD adjudication is considerable in routine practice (12). For patients whose disease is genuinely racing ahead, waiting out the confirmation interval can delay a switch to salvage therapy—the opposite of the safety net iRECIST intends (15, 16). Automated lesion measurement may eventually reduce reader variability in this workflow (17), but automation of measurement does not address the kinetic gap discussed below.

### 3.2 Atypical response patterns: imaging features, clinical context, and diagnostic pitfalls

Table 1 summarizes the four patterns clinicians most need to separate. On CT, pseudoprogression looks like progression: target lesions enlarge, and new lesions can appear (18). The first clue is often clinical—the patient feels stable or better. On 18F-FDG PET/CT, a dissociation between size and metabolism (enlargement with stable or falling standardized uptake value, SUV) points to pseudoprogression rather than true growth (18, 19). Across trials, roughly one patient in six who continues therapy beyond a RECIST 1.1 PD readout ultimately proves to have pseudoprogression, and survival in this group is substantially better than in true progressors (13, 20).

**Table 1:** Comparison of atypical response patterns to immunotherapy from imaging and clinical perspectives.

Pattern	CT appearance	Functional imaging	Clinical context	Prognosis	Management implication
Pseudoprogression	Enlargement or new lesions at first assessment, followed by regression without change of therapy (13, 18)	FDG: size–metabolism dissociation (enlargement with stable or falling SUV) (18)	Stable or improving symptoms; ∼6% of ICI-treated patients (95% CI 5%–7%) (2)	Favorable; longer OS than true progression (e.g., 29.9 vs. 8.0 months) (13)	Continue ICI; confirm with early repeat imaging (iRECIST iUPD → iCPD pathway) (5)
True (conventional) progression	Progressive enlargement or new lesions; no later regression (1)	FDG: concordant increase in size and metabolism (18)	Worsening symptoms, declining performance status	Poor	Switch therapy if clinically indicated; no confirmatory delay required
Hyperprogressive disease	Rapid, disproportionate enlargement shortly after treatment start (21)	FDG: high but nonspecific uptake; no validated metabolic criterion (18)	Often rapid clinical decline; incidence ∼5%–10% of treated patients (7)	Very poor (3)	Kinetic confirmation required (TGRpost/TGRpre ≥ 2 with pre-baseline scan); if confirmed, stop ICI and switch strategy (21, 22)
Dissociated response	Concurrent shrinkage of some lesions and growth of others (4)	FDG: mixed metabolic response across lesions (18)	Variable; depends on the burden of progressing sites	Intermediate; better than uniform progression (4)	Lesion-by-lesion strategy: local therapy to progressing sites, continue systemic treatment (4, 18)

FDG, fluorodeoxyglucose; ICI, immune checkpoint inhibitor; iCPD, immune confirmed progressive disease; iUPD, immune unconfirmed progressive disease; OS, overall survival; SUV, standardized uptake value; TGR, tumor growth rate.

**Figure 2:**
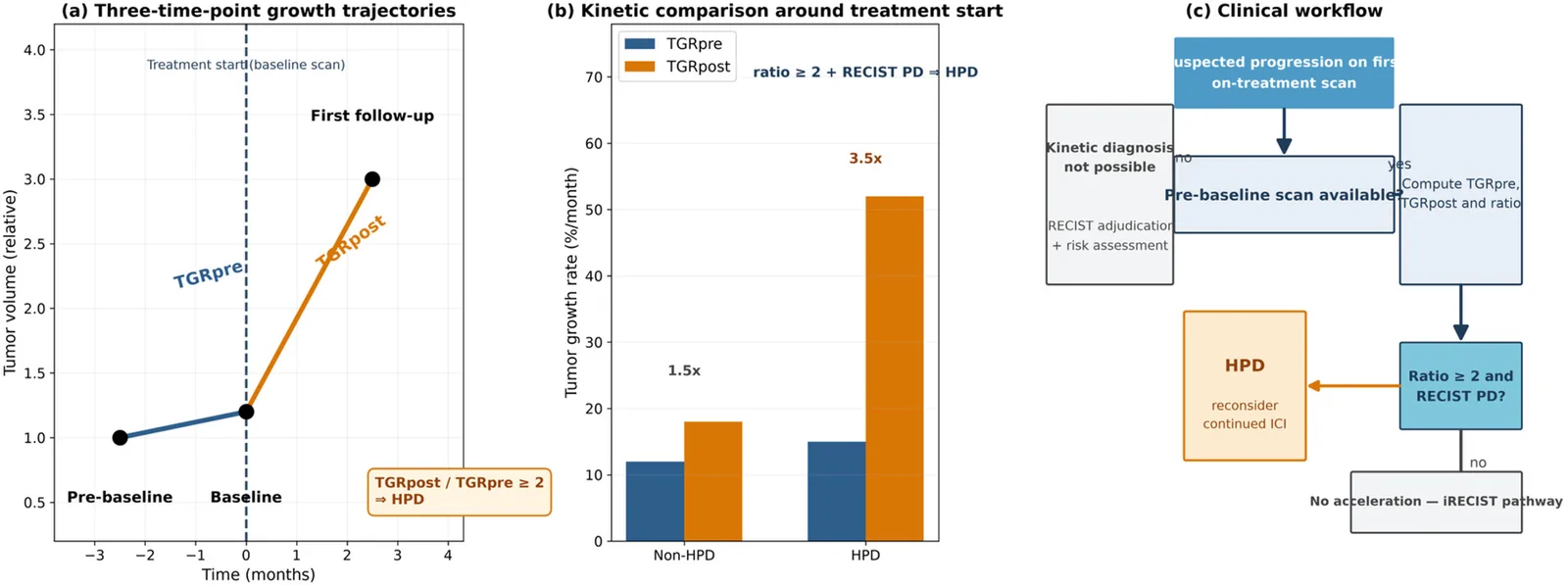
Longitudinal framework for evaluating suspected hyperprogressive disease (HPD). **(a)** Three-time-point acquisition (pre-baseline, baseline, first on-treatment follow-up) establishes pre- and on-treatment growth trajectories. **(b)** The ratio of post-treatment to pretreatment tumor growth rate (TGRpost/TGRpre), combined with RECIST-defined progressive disease, discriminates HPD from non-accelerated progression. **(c)** Clinical workflow integrating kinetic assessment with iRECIST adjudication; without a pre-baseline scan, kinetic diagnosis is not possible and only risk assessment can be offered.

HPD is a different problem. Over a single short interval, a rapidly enlarging HPD lesion is indistinguishable from ordinary PD; the difference lies in the slope. HPD is defined by acceleration of growth relative to the pretreatment trajectory: under an exponential volume model, the tumor growth rate (TGR) is computed as 100 × [exp[3 × ln(Sₜ/S₀)/(t − t_0_)] − 1], and HPD is typically declared when TGR after treatment is at least twice the pretreatment TGR in a patient who also meets RECIST-defined PD (21, 22). Because the definition is kinetic, it requires three time points: pre-baseline (before the treatment decision), baseline (immediately before the first dose), and first on-treatment follow-up (Figure 2). Thresholds vary across studies, new lesions complicate the arithmetic, and many retrospective datasets simply lack the pre-baseline scan—which means a definitive HPD label is unattainable for a large fraction of archival patients. Some groups have therefore operationalized HPD without a pre-baseline scan, using short time-to-treatment failure combined with a marked increase in tumor burden; these pragmatic definitions trade kinetic precision for feasibility and do not measure acceleration directly (3, 21).

Dissociated responses, affecting about 3.3% of patients on combination immunotherapy, are the radiographic signature of a heterogeneous immune microenvironment: some lesions respond while others progress (4). Summed-diameter assessment erases this signal entirely (18). Management often requires lesion-by-lesion judgment—local therapy to a progressing site while systemic treatment continues.

## 4 Multimodal imaging of the tumor immune microenvironment

### 4.1 Functional and molecular imaging

18F-FDG PET/CT remains the most accessible functional modality for immunotherapy monitoring. Metabolic change—a falling SUV—often precedes anatomical shrinkage by weeks, and the dissociation between size and metabolism is one of the earliest clues to pseudoprogression (23). Beyond FDG, a growing set of immuno-PET tracers targets specific molecular players in the immune response (24, 25). [89Zr]Zr-atezolizumab couples a full-length anti-PD-L1 antibody to zirconium-89 and maps PD-L1 distribution across the whole body; heterogeneous uptake among lesions has been linked to divergent outcomes, though the tracer remains investigational (26). A 2026 feasibility study extended this approach to metastatic triple-negative breast cancer (27). CD8-targeted probes such as [89Zr]Zr-Df-IAB22M2C (crefmirlimab berdoxam) have reached phase I evaluation and can visualize CD8+ T-cell distribution across metastatic sites—a direct readout of immune infiltration that radiomics can only approximate (28).

The imaging–immunology interface is best illustrated by correlations between radiomic features and specific immune-cell populations. CT texture features—entropy, uniformity, and gray-level co-occurrence matrix (GLCM) contrast in particular—have been linked to CD8+ T-cell density, with higher entropy (greater image heterogeneity) corresponding to a more inflamed phenotype and low-entropy, homogeneous textures to immune-desert lesions (29). Peritumoral radiomic features on contrast-enhanced CT reflect the vascular and stromal remodeling that accompanies T-cell trafficking; a CT-TIME signature combining intratumoral and peritumoral features with RNA-sequencing data classified T-cell-inflamed vs. non-inflamed phenotypes with AUCs of 0.78–0.85 across independent cohorts (30). MRI-based texture analysis in glioma, by contrast, has identified radiomic patterns that track tumor-associated macrophage density rather than T-cell abundance, a reminder that different imaging feature families capture different immune compartments (31). A critical gap persists across this literature: few studies validate radiomic–immune-cell associations against spatially co-registered biopsies, leaving open the possibility that the imaging signal reflects vascular, stromal, or necrotic change rather than immune infiltration itself.

### 4.2 Direct and indirect assessment of tumor-infiltrating lymphocytes

Indirect TIL characterization through radiomics integrates high-throughput feature extraction with machine learning (32, 33). It is scalable—whole tumors and all metastatic sites can be profiled noninvasively—but the link between a texture feature and a specific immune-cell type is statistical, not causal (33). Direct characterization visualizes immune-cell populations with molecular specificity; CD8-targeted immuno-PET is the most advanced example, although tracer uptake does not equal cytotoxic function, since activated and exhausted T cells both express CD8 (28). Spatial organization matters too: the inflamed-vs.-excluded phenotype, defined by the position of TILs relative to the tumor margin, carries prognostic information that bulk radiomic features can miss (23, 31). AI-based spatial analysis of MRI or PET images is beginning to infer such phenotypes without tissue sampling, but multicenter validation is scarce.

## 5 AI and radiomics: risk prediction, not kinetic diagnosis

### 5.1 A distinction the evidence requires

One distinction must be made explicit before any study is discussed. HPD is defined by acceleration of growth relative to a pretreatment trajectory (21, 22). A model trained on a baseline scan, or even on baseline and first follow-up scans, does not measure acceleration; it estimates the probability that a patient belongs to a fast-growing phenotype. That is risk prediction. Kinetic diagnosis—the statement that growth has accelerated after treatment began—is a different claim and requires the pre-baseline time point. Almost every model reviewed below is a risk predictor. We therefore describe these tools as triage aids: they can flag patients for closer monitoring or enrich clinical trials, but they cannot diagnose HPD, and presenting them as diagnostic is a category error (7, 34). The corollary is architectural: if training labels encode only baseline and post-treatment information, the model cannot learn acceleration even in principle; only labels that incorporate a pre-baseline growth interval can carry kinetic information (7, 30).

### 5.2 Radiomics in immunotherapy: workflow and applications

A reproducible radiomics pipeline depends on standardized acquisition, segmentation, feature extraction, and modeling. The Image Biomarker Standardisation Initiative (IBSI) published reference values for 169 standardized radiomic features (from an initial set of 174) and a framework for software-level verification (35). IBSI compliance does not remove biological variability, but it ensures that cross-institutional differences reflect disease biology rather than implementation artifacts (36).

Clinical applications now span several tumor types. In biliary tract cancer, a nomogram combining lymph-node radiomic features with clinical variables predicted immunotherapy response with strong discrimination (37). In nasopharyngeal carcinoma, MRI-based radiomics outperformed the PD-L1 combined positive score as a predictor of objective response (38). A DeepSurv model integrating intratumoral and peritumoral vascular features with clinical data stratified progression-free survival in NSCLC (39), and short-term peri- and intratumoral radiomics predicted response in advanced NSCLC (40). These examples mark a shift from single-molecule biomarkers toward multivariate image-derived signatures—a welcome development that nonetheless demands rigorous external validation before clinical use.

A caution applies to HPD specifically. Models trained on baseline and post-treatment scans cannot separate kinetic acceleration from simple progression unless the training labels include a pre-baseline growth interval; without that frame of reference, the model may learn tumor burden, time-to-treatment-failure, or other proxies rather than true growth acceleration (7, 30). The pan-cancer CT-TIME signature and similar tools are best framed as triage aids that flag atypical enlargement for expedited clinical review, not as diagnostic classifiers of HPD (30).

### 5.3 Deep learning and longitudinal models

Deep learning replaces handcrafted features with learned representations. Convolutional neural networks (CNNs)—ResNet, DenseNet, and EfficientNet variants—apply local spatial filters that capture edges, textures, and morphology in a hierarchical fashion. Vision transformers (ViTs) divide the image into patches and use self-attention to model relationships across the whole image, a property that may help capture long-range tissue interactions in heterogeneous tumors (41). Hybrid CNN-transformer architectures try to keep local boundary detail while adding global context; their memory demands in 3D imaging are substantial, often forcing patch-level sampling or gradient checkpointing.

Training these models in the immunotherapy setting follows a familiar playbook. Transfer learning from large natural-image or non-oncologic CT databases initializes weights and compensates for small target cohorts. Data augmentation—random rotations, elastic deformations, intensity shifts—improves robustness to acquisition variability. Class imbalance, common for rare outcomes such as HPD (5%–10% of treated patients), is handled with oversampling, synthetic minority augmentation, or weighted loss functions. Evaluation should extend beyond the area under the receiver operating characteristic curve (AUC) to sensitivity, specificity, and predictive values at clinically relevant thresholds, calibration (Brier score, integrated calibration index), and net reclassification improvement; survival endpoints call for concordance indices and time-dependent AUC (10, 11). Internal validation alone—random splits or k-fold cross-validation—overstates performance; held-out data from a different institution, vendor, or region is the minimum standard for any generalizability claim (34).

Longitudinal modeling adds a temporal dimension. Serial CT or PET scans can be encoded as token sequences and processed by a transformer with temporal positional embeddings, which handles irregularly spaced visits and reveals which time points drive the prediction (42). Delta-radiomics—the study of change in radiomic features between time points—is a simpler but effective longitudinal strategy: in NSCLC and melanoma, delta-radiomics models outperformed single-time-point counterparts for response and survival prediction (43, 44). An ensemble CT deep-learning model (Deep-CT) trained on 976 NSCLC patients reached an OS C-index of 0.75 when combined with clinical variables, vs. 0.70 for clinical data alone (34). Selective state-space models such as Mamba have been proposed as linear-complexity alternatives to transformer self-attention for long image sequences; we are not aware of any published immunotherapy CT or PET cohort validating them, and they are mentioned here only as a direction to watch, not as evidence.

Multimodal fusion—combining CT, PET, and clinical or genomic data—can be implemented at three levels. Early fusion concatenates raw features before modeling; it captures cross-modal interactions but is fragile when a modality is missing. Joint fusion (shared latent representations, cross-attention) offers richer interactions at the price of requiring large paired datasets. Late fusion trains separate models per modality and combines their outputs; it is modular and tolerates missing data but may lose fine-grained cross-modal patterns (45). A practical example is the fusion nomogram combining contrast-enhanced CT radiomics, deep features, and clinical variables to predict durable clinical benefit in advanced NSCLC (AUC: 0.89) (46).

### 5.4 What do the reported performance gains mean clinically?

A C-index gain from 0.70 to 0.75, as reported when imaging was added to clinical variables in the Deep-CT model (34), is statistically respectable but clinically modest: at the level of an individual patient it does not, by itself, justify starting, stopping, or switching therapy. Ranking metrics describe how well a model orders patients; they do not measure the net benefit of acting on its output. A model can rank patients correctly and still fail to improve decisions if calibration is poor or if the actions it suggests are not feasible or acceptable to clinicians (10, 11). Decision-curve analysis, net reclassification improvement, and calibration testing at clinically relevant thresholds are therefore not optional extras, and their absence from most published studies is itself a finding. Measured against the existing standard of care—serial CT, iRECIST adjudication, and clinical judgment—no imaging-AI model has yet been shown, in a prospective study, to change management in a way that improves survival, spares futile treatment, or reduces premature discontinuation. That is the comparison that matters, and it has not been made.

## 6 Clinical translation: data, interpretability, and ethics

### 6.1 Standardized longitudinal datasets

Heterogeneity of imaging data—vendors, reconstruction kernels, contrast protocols, dose settings—is a known cause of performance degradation at new sites (48). Collaborative infrastructures such as EUCAIM, which aggregates European cancer imaging data through centralized and federated architectures, show the way toward adequately powered, diverse cohorts (49), and flexible metadata models such as MINDS ease the integration of imaging, clinical, genomic, and pathology data into machine-learning-ready datasets (50). Protocol standardization per QIBA guidelines reduces but does not eliminate acquisition variability. Complementary approaches include feature-level harmonization (ComBat, adversarial domain classifiers) and image-level translation (VAE-GAN, CycleGAN), both of which improved cross-scanner consistency in MRI and PET studies (36, 51, 52). The caveat is that generative harmonization can alter small lesions or quantitative uptake values; evaluation pipelines must therefore include lesion-preservation tests, phantom data, and external-site validation.

**Figure 3:**
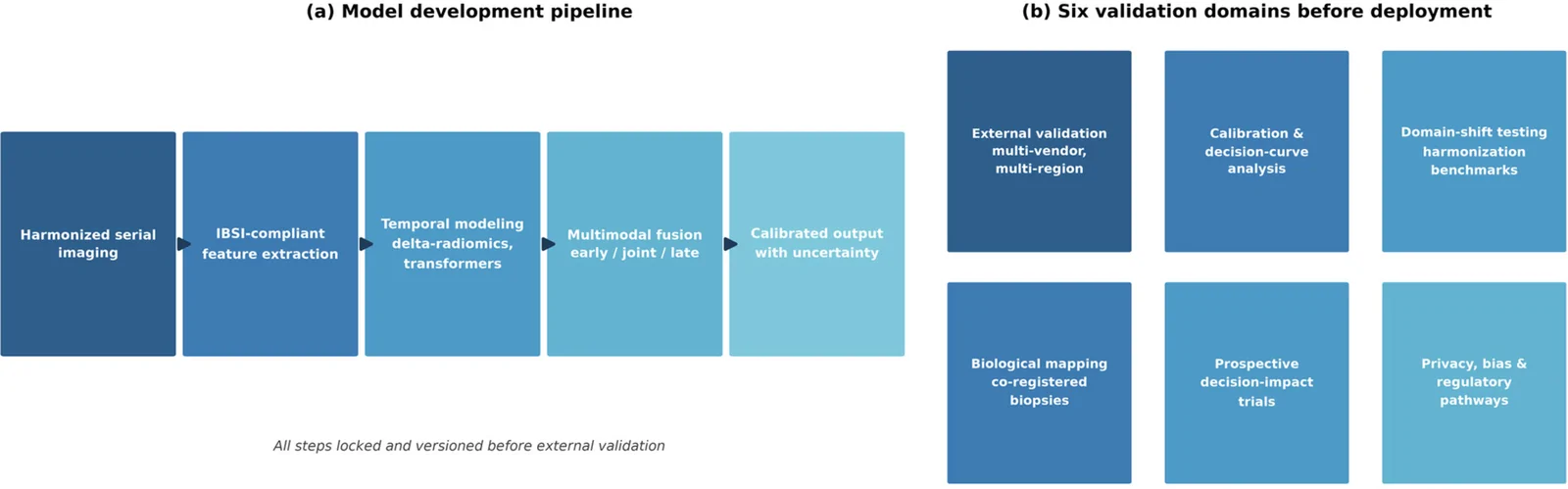
Framework for translating longitudinal multimodal imaging-AI into clinical practice. **(a)** Model development pipeline: harmonized serial imaging, IBSI-compliant feature extraction, temporal modeling, multimodal fusion, and calibrated output. **(b)** Six validation domains that must each be addressed before clinical deployment.

For HPD modeling specifically, pre-baseline imaging is a structural requirement that no algorithm can circumvent. Reports should publish scan windows, lesion-selection rules, TGR formulas, missing-data patterns, and inter-reader agreement statistics. Transparent reporting per TRIPOD + AI (10) is the least a field can demand of itself. Figure 3 summarizes the development pipeline and the six validation domains that must each be addressed before deployment.

### 6.2 Interpretability and trust

Explainability methods such as Grad-CAM (which highlights the image regions driving a prediction) and SHAP (which attributes importance to individual features) are useful debugging tools, but saliency does not prove that a model learned a clinically meaningful mechanism (53, 54). A more rigorous path is biological mapping: prespecified imaging features are tested against spatially and temporally matched biological measurements—perfusion and hypoxia-sensitive PET/MRI parameters against hypoxia markers, peritumoral radiomic features against angiogenesis gene-expression signatures, longitudinal immuno-PET signal changes against CD8 density on sequential biopsies (33). These mappings are falsifiable: a model whose imaging signal fails to track the corresponding biology should be set aside regardless of its AUC.

### 6.3 Ethical and regulatory dimensions

Privacy is the most immediate concern. Longitudinal imaging and multi-omics data are highly identifiable; federated learning, which shares only model updates rather than data, reduces but does not eliminate re-identification risk, and privacy guarantees must be technically verifiable rather than asserted (55). Algorithmic bias is subtler but equally serious: cohorts drawn from a single demographic group, region, or scanner vendor can degrade sharply when applied elsewhere, and disparities in healthcare access mean the technology may reach the well-resourced centers that need it least. TRIPOD + AI now requires diversity reporting and subgroup analyses (10). Liability frameworks for AI-assisted decisions remain unsettled: if a model misclassifies pseudoprogression as progression and treatment stops, responsibility could fall on the physician, the deploying institution, or the developer. Labeling AI outputs as assistive rather than determinative, combined with transparent uncertainty quantification, is a step toward clarity (54). Regulators including the FDA and NMPA have issued draft guidance for software as a medical device, but dedicated pathways for continuously learning models are still under development; prospective trials measuring both model performance and downstream patient outcomes are the only route to resolving these tensions in practice (55). Major limitations of existing imaging-response frameworks and representative immunotherapy AI-imaging investigations are outlined in Tables 2, 3.

**Table 2:** What major imaging-response frameworks can and cannot establish about hyperprogressive disease.

Framework	Required imaging	Key rule	Strength	HPD limitation
RECIST 1.1 (1)	Baseline + follow-up	PD by size increase or new lesion	Simple, reproducible	Does not separate HPD from conventional PD or pseudoprogression
iRECIST (5, 15)	Baseline + first assessment; confirmatory scan after iUPD	iUPD → iCPD requires progression on confirmatory scan	Reduces premature termination during pseudoprogression	HPD and conventional PD both satisfy iCPD; not designed for kinetic diagnosis
PERCIST/immune-metabolic (18, 19)	Baseline + serial PET	Metabolic progression (SUV) and/or new avid lesions	Adds functional dimension before size change	Inflammation confounds uptake; metabolic progression ≠ kinetic acceleration
Kinetic HPD definitions (21, 22)	Pre-baseline + baseline + post-treatment	RECIST PD + TGRpost/TGRpre ≥ 2	Direct test of growth acceleration	Retrospectively infeasible without pre-baseline; thresholds vary

HPD, hyperprogressive disease; iCPD, immune confirmed progressive disease; iUPD, immune unconfirmed progressive disease; PD, progressive disease; TGR, tumor growth rate.

**Table 3:** Representative imaging-AI studies in immunotherapy and the limits of their clinical claims.

Study	Cohort and imaging	Endpoint/model	Reported performance	What the study does NOT establish
Vaidya et al. (7)	Advanced NSCLC; *n* = 109; pretreatment CT + vasculature	HPD risk; machine-learning classifier	AUC: 0.85–0.96	Does not diagnose HPD; risk flag only (19 HPD cases)
Saad et al. (34)	Advanced NSCLC; *n* = 976; pretreatment CT + clinical	OS/PFS; ensemble Deep-CT	OS C-index 0.70 → 0.75	Survival prediction ≠ response-pattern classification
Bernatowicz et al. (30)	Pan-cancer; *n* = 428; 1,360 tumors; CECT radiomics + RNA-seq	CT-TIME inflamed phenotype	AUC: 0.78–0.85	Biological signature, not kinetic HPD diagnosis
Han et al. (43)	NSCLC; 2 centers; *n* = 179; baseline + 6–8-week CT	PFS/OS; delta-radiomics	C-index 0.60 (PFS)	Modest transport performance; no HPD endpoint
Chen et al. (44)	Metastatic melanoma; *n* = 50; pre- + post-treatment CT	Response; automated multidimensional delta-radiomics	AUC: 0.86 → 0.73 (test)	Small cohort; endpoint is response, not HPD
Zhu et al. (46)	Advanced NSCLC; *n* = 201; CECT + ResNet-34 + clinical	Durable clinical benefit; nomogram	AUC: 0.84–0.89	Durable benefit ≠ early response pattern
Ye et al. (47)	Resectable NSCLC (neoadjuvant); *n* = 534; pre- + post-treatment CT	Pathologic complete response; longitudinal deep learning	AUC: 0.87 (external validation)	Neoadjuvant pCR ≠ metastatic ICI response or HPD

Metrics are not directly comparable across rows because cancer types, endpoints, prevalence, and validation designs differ. AUC, area under the ROC curve; C-index, concordance index; CECT, contrast-enhanced CT; HPD, hyperprogressive disease; ICI, immune checkpoint inhibitor; NSCLC, non-small cell lung cancer; OS, overall survival; pCR, pathologic complete response; PFS, progression-free survival.

## 7 Future directions: toward an imaging-immunity biomarker atlas

### 7.1 Multi-omics integration: a panoramic view

Converging radiomics with genomics, transcriptomics, proteomics, and spatial omics promises to map imaging phenotypes to their molecular drivers, via early, joint, or late fusion strategies with their respective trade-offs between interaction depth and robustness to missing data (45). In lung adenocarcinoma, CT radiomic subtypes have been linked to immune-infiltration profiles and survival (56); in hepatocellular carcinoma, a clinical-radiomics model tracked MHC-I expression, CD8 gene signatures, and TP53 mutation status (57). Spatial transcriptomics and proteomics add a tissue-context dimension that bulk sequencing cannot provide, and linked to co-registered imaging they could anchor the biological mapping discussed in Section 6.2 (58).

### 7.2 Personalized therapy and dynamic resistance monitoring

Early identification of non-responders and of patients at risk of hyperprogression could enable earlier treatment adjustments—but without a valid pretreatment trajectory, the appropriate output is a calibrated risk estimate, not an HPD diagnosis (7, 59). Prospective trials with prespecified early imaging windows and model-informed reassessment protocols are needed to show that such tools improve outcomes without increasing premature discontinuation during pseudoprogression. Combination regimens—immunotherapy with radiotherapy, targeted agents, or anti-angiogenic drugs—add further complexity: imaging can monitor abscopal effects after radiotherapy, vascular normalization by anti-angiogenic agents, and early metabolic changes signaling synergy or resistance (23). Longitudinal radiomic trajectories may capture the transition from an inflamed to an excluded phenotype under treatment pressure, offering a window into acquired resistance (56, 60).

### 7.3 Research priorities: a staged roadmap

Near term (1–3 years): the single highest-yield investment is prospective, multicenter collection of standardized longitudinal datasets that include pre-baseline imaging wherever kinetic endpoints are intended. Parallel efforts should target IBSI compliance and domain-adaptation benchmarking across vendors. Existing radiomics and deep-learning models should undergo independent external validation with locked code and transparent reporting per TRIPOD + AI (10), with calibration and decision-curve analysis as mandatory components.

Medium term (3–5 years): biological mapping studies pairing longitudinal imaging with spatially co-registered biopsies, spatial transcriptomics, and multiplex immunohistochemistry can establish whether radiomic signals correspond to specific immune-cell populations or functional states. Multi-omics fusion models should be validated in prospective cohorts with intentional demographic and geographic diversity. Decision-impact trials—randomizing clinicians to model-assisted vs. standard assessment—are the only design that can establish whether AI tools change management in ways that improve patient outcomes (10, 11).

Long term (5–10 years): a multidimensional imaging-immunity biomarker atlas, linking longitudinal imaging phenotypes to spatially resolved immune-cell distributions and molecular pathways, could serve as the backbone of precision immuno-oncology—provided the field sustains international collaboration, develops regulatory frameworks for continuously learning models, and builds reimbursement models that recognize the value of noninvasive dynamic assessment. Architectural novelty, whether transformers, state-space models, or their successors, will not substitute for correct endpoint construction, complete longitudinal data, and reproducible multicenter performance.

## 8 Conclusion

Immunotherapy has forced a rethinking of what counts as treatment response. Static size-based criteria are being supplemented, and in some settings supplanted, by immune-adapted frameworks, functional and molecular imaging, and AI-driven longitudinal analysis. The evidence reviewed here supports a specific and sober conclusion: these tools can stratify risk and generate biological hypotheses, but none has reached the validation level required for routine decision-making, and none diagnoses HPD. HPD is the instructive case: the requirement for pre-baseline imaging is absolute, and no analytical architecture, however sophisticated, can reconstruct a missing growth trajectory from an incorrectly defined endpoint.

Progress depends less on any single technology than on a coordinated set of practices: prospective data collection that includes pre-baseline imaging when kinetic endpoints are intended; endpoint definitions that separate morphologic progression from kinetic acceleration; IBSI-compliant feature extraction; external validation across vendors and populations; and prospective decision-impact trials. Ethics—privacy, bias, accountability—must be addressed alongside technical performance, not after it. The imaging-immunity biomarker atlas remains aspirational but achievable, if the field holds imaging-AI to the same evidentiary standards it applies to the therapeutics it seeks to guide.
